# Screening for Hypertrophic Obstructive Cardiomyopathy in Patients With Panic Disorder: A Case Report

**DOI:** 10.7759/cureus.31811

**Published:** 2022-11-23

**Authors:** Thomas Bonitz, Luke Scypinski, Justin Chin, Christine M Lomiguen

**Affiliations:** 1 Medical Education, Lake Erie College of Osteopathic Medicine, Erie, USA; 2 Family Medicine, LifeLong Medical Care, Richmond, USA; 3 Family Medicine, LECOM (Lake Erie College of Osteopathic Medicine) Health Millcreek Community Hospital, Erie, USA; 4 Pathology, Lake Erie College of Osteopathic Medicine, Erie, USA

**Keywords:** aha/aca, hocm screening, echocardiogram (echo), electrocardiogram (ecg/ekg), sudden cardiac death (scd), left ventricular hypertrophy (lvh), ventricular tachycardia (vt), left ventricular outflow obstruction (lvot), panic disorders, hypertrophic obstructive cardiomyopathy (hocm)

## Abstract

Hypertrophic obstructive cardiomyopathy (HOCM) is a cardiovascular disease that is widely recognized as an important cause of various cardiovascular pathologies. Passed through an autosomal dominant inheritance pattern, mutations can result in cardiac dysfunction that can manifest in dyspnea, exercise intolerance, and sudden death. Panic disorder can present similarly to HOCM; however, precautions and treatment differ significantly. Here, we present a case of a 56-year-old male with a history of panic disorder who presented to the emergency department with recurrent episodes of palpitations, lightheadedness, and dyspnea, and who was subsequently hospitalized due to new ventricular tachyarrhythmia and diagnosed with HOCM. This case highlights the importance of detailed history taking, follow-up of chronic symptoms, and consideration of genetic screening for HOCM in patients with panic disorder.

## Introduction

Hypertrophic obstructive cardiomyopathy (HOCM) is a cardiovascular disease that is widely recognized as an important cause of ventricular tachyarrhythmias, sudden cardiac death, heart failure, and atrial fibrillation with associated thromboembolism [1]. HOCM is a genetic disorder with an autosomal dominant inheritance pattern in which 14 genes and more than 1,400 mutations have been identified [2]. These mutations can lead to diastolic dysfunction and left ventricular outflow tract (LVOT) obstruction, which can cause an increase in wall tension, increased myocardial oxygen consumption, impaired systolic performance, and mitral insufficiency. Screening is typically predicated on identifying a family history of HOCM and confirmed with "dagger-like" Q waves and large precordial voltages on an electrocardiogram (EKG) and/or increased left ventricular wall thickness (≥15 mm) on an echocardiogram [3]. Without medical management and intervention, HOCM can result in sudden cardiac death, which is one of the most common causes of cardiac-related mortality in children and adolescents [4]. While one of the most commonly inherited cardiovascular diseases, its presentation is often nonspecific and may be omitted during the workup of palpitations or shortness of breath [5]. 

In comparison with HOCM, panic disorder can present similarly, with additional symptoms of hot flashes, nausea, and paresthesia. Panic disorder does have distinct symptoms such as derealization or depersonalization, dry mouth, urge to urinate or defecate, accompanied worrying, and environmental or psychological triggers that precede panic attacks [6]. Panic disorders are much more common than HOCM and can impact up to 5% of individuals at some point during their lifetimes [7]. Short- and long-term treatments can include behavioral counseling, cognitive behavioral therapy, psychotherapy, anxiolytics, and selective serotonin reuptake inhibitors. According to the Diagnostic and Statistical Manual of Mental Disorders-V (DSM-5), the diagnosis of panic disorder is given only when organic physiological or concomitant mental health disorders are ruled out and may include thyroid dysfunction, epilepsy, substance use, or arrhythmias [8]. While HOCM and panic disorder have similar presentations, their management and treatment vary greatly.

Here, we present a case of a 56-year-old male with a reported history of panic disorder who presented with recurrent episodes of palpitations and shortness of breath, and who was subsequently found to have HOCM. This case highlights the importance of thorough history taking, challenging anchoring bias, and encouraging clinicians to consider genetic screening for HOCM in patients with panic disorder with inconclusive cardiac testing.

## Case presentation

A 56-year-old Caucasian male with a history of panic disorder presented to the emergency room following an episode of palpitations, lightheadedness, dyspnea, shortness of breath, diaphoresis, muscle weakness, fatigue, difficulty speaking, bilateral lower extremity paresthesia, and feelings of impending doom lasting for several minutes at a time. Prior to this event, he had a 10+ year history of similar episodes, but the patient subjectively noted that they were less severe, tended to correlate with high levels of stress every two to three months, and resolved after rest. He was previously diagnosed with panic attacks but never had a behavioral or cardiac workup due to financial and time constraints. His Generalized Anxiety Disorder-7 (GAD-7) and Patient Health Questionnaire-9 (PHQ-9) results were equivocal with scores of three and four, respectively. Surgical history and social history were noncontributory. There was no known psychiatric history for the patient and family during the initial intake. His family history was initially positive for various cardiac pathologies, including atrial fibrillation and multiple strokes with his father and atrial fibrillation, coronary artery disease, and multiple myocardial infarctions with his mother. Further genealogical history was not available. 

Vitals were significant for tachycardia with a heart rate of 110 beats per minute, normotensive at 123/78, and saturating at 98% on room air. Physical examination was benign, with tachycardia on auscultation but a regular rhythm and no murmur noted. The EKG revealed ventricular tachycardia, for which he was given intravenous adenosine of 6 mg and intravenous amiodarone of 150 mg. Cardiac troponins were mildly elevated (0.30, 0.45, 0.31; normal range 0-0.04), with normal electrolytes, a complete blood count, and a lipid panel. Given his family cardiac history, the patient was administered oral aspirin of 325 mg and underwent cardiac catheterization, which revealed no obstruction of the coronary vessels. Cardiology was consulted and recommended a transthoracic echocardiogram, which revealed left ventricular hypertrophy suggestive of being the cause of the aberrant ventricular tachyarrhythmias. In consultation with cardiothoracic surgery, an implantable cardioverter-defibrillator (ICD) was placed, and the patient was started on warfarin and continued amiodarone.

Upon outpatient follow-up, a repeat echocardiogram was performed, which revealed left ventricular hypertrophy with mild subaortic LVOT obstruction. Given the constellation of symptoms and cardiac testing results, the patient underwent genetic testing for common HOCM gene mutations, which revealed that he had the cardiac beta-myosin heavy chain (*MHY7*) gene mutation found in HOCM. Six months status post ICD placement, he was switched from amiodarone to metoprolol. He was tapered off metoprolol and warfarin in the following year. Subsequent genetic testing of the patient’s siblings revealed three out of five with *MHY7* gene mutations with varying degrees of obstruction and left ventricular hypertrophy. The patient’s children declined genetic testing at this time, but they plan to do so in the future.

## Discussion

Patients with HOCM initially have no or minor symptoms, and screening occurs due to known family history, murmur on auscultation, or abnormal EKG. In the event of a prolonged history of untreated HOCM, classical symptoms of dyspnea on exertion, fatigue, atypical or anginal chest pain, and palpitations can occur [9]. The prevalence of HOCM has been classically estimated to affect 1:500 people in the general population; however, new data with gene sequencing and carriers now estimate closer to 1:200 [10, 11]. In comparison, panic disorder is much more common, but it is a diagnosis of exclusion as psychiatric conditions can often mimic other organic diseases [12, 13]. In this case, it is unclear whether the diagnosis of panic disorder was warranted; however, the patient satisfied the American Heart Association/American College of Cardiology guidelines for the diagnosis and treatment of HOCM (Table 1) [2]. While the patient’s family history was suspicious for cardiac pathologies, the lack of confirmed HOCM and the advent of newer screening and imaging modalities likely precluded earlier diagnosis. The availability of outpatient EKG and point-of-care echocardiogram has contributed to the early diagnosis of HOCM [14, 15]. It is critical that physicians rule out HOCM before defaulting to less deadly differential etiologies of the patient’s symptoms.

**Table 1:** Ten recommendations for HOCM management and treatment. HOCM: hypertrophic obstructive cardiomyopathy; ICD: implantable cardioverter-defibrillator.

Recommendations for HOCM management and treatment	
1.	Shared decision-making is important when discussing HOCM management.
2.	Multidisciplinary HOCM treatment teams can help optimize care.
3.	Genetic counseling is important for patients and their families.
4.	Cardiac imaging, particularly with an echocardiogram, is important in confirming HOCM.
5.	Evaluation of the need for ICD placements needs to occur regularly as risk factors evolve.
6.	ICD placement in children requires multidisciplinary discussions in HOCM treatment teams.
7.	Septal reduction therapies should be considered in drug-resistant HOCM.
8.	Oral anticoagulation should be considered in patients with HOCM and atrial fibrillation.
9.	Heart failure symptoms in HOCM should be treated in the same manner as heart failure.
10.	Exercise has a beneficial role in decreasing cardiac events in HOCM patients.

Genetic testing has become the mainstay in diagnosing HOCM prior to patients developing symptoms. While not applicable to the case presented, genetic testing was recommended to the patient's children given the variable penetration and expression of genes associated with HOCM [16]. Initially, genetic testing for HOCM was performed using linkage analysis and candidate gene sequencing, which was both time-consuming and expensive. Screening for HOCM entails looking for the most involved genes, which include *MYH7*, cardiac myosin binding protein-C (*MYBPC3*), cardiac troponin T (*TNNT2*), and cardiac troponin I (*TNNI3*), with the first two accounting for almost 80% of all HOCM patients [17]. *MYH7* and *MYBPC3 *contain sequences that encode for sarcomeres or sarcomere-associated proteins, in which next-generation sequencing (NGS) can rapidly analyze for mutations in these genes [18, 19]. Patients with suspected genetic etiologies of left ventricular hypertrophy may also have mutations that are separate from HOCM and can include Fabry disease, Noonan syndrome, and other lysosomal storage disorders [20]. With NGS, screening protocols may be developed and implemented into clinical practice when patients have a family history of heart disease or have experienced common symptoms associated with HOCM or panic disorder, especially as genomic and personalized medicine becomes more advanced.

## Conclusions

HOCM is a cardiac condition that can masquerade as anxiety and panic disorder. Clinical history and appropriate screening modalities are crucial to diagnosis, and genetic testing can be used for confirmation and to screen asymptomatic relatives. As seen in this case, diagnosis delays and adverse outcomes can occur when HOCM is not considered in the differential diagnosis. Future studies can build upon creating a risk-stratification tool that utilizes family history and cardiac findings for patients with panic disorder to rule out HOCM.
